# Direct observation of atomic-scale fracture path within ceramic grain boundary core

**DOI:** 10.1038/s41467-019-10183-3

**Published:** 2019-05-08

**Authors:** Shun Kondo, Akihito Ishihara, Eita Tochigi, Naoya Shibata, Yuichi Ikuhara

**Affiliations:** 1 Institute of Engineering Innovation, School of Engineering, The University of Tokyo, Bunkyo, Tokyo 113-8656 Japan; 2 Center for Elements Strategy Initiative for Structural Materials, Kyoto University, Kyoto, 606-8501 Japan; 3 Nanostructures Research Laboratory, Japan Fine Ceramics Center, Nagoya, Aichi 456-8587 Japan

**Keywords:** Ceramics, Transmission electron microscopy, Atomistic models

## Abstract

In fracture processes, grain boundaries behave as preferential paths for crack propagation. These grain boundary fractures proceed by the atomic-bond rupture within the grain boundary cores, and thus grain boundary structures have crucial influence on the fracture properties. However, the relationship between grain boundary structures and atomic fracture processes has been a matter of conjecture, especially in the case of dopant-segregated grain boundaries which have complicated local structures and chemistries. Here, we determine the atomic-bond breaking path within a dopant-segregated Al_2_O_3_ grain boundary core, via atomic-scale observations of the as-fractured surface and the crack tip introduced by in situ nanoindentation experiments inside a transmission electron microscope. Our observations show that the atomic fracture path is selected to produce less coordination-deficient oxygen polyhedra of dopant cations, which is rationalised using first-principles calculations. The present findings indicate that the atomic coordination geometry at the grain boundary core affects the fracture processes.

## Abstract (web summary)

Cracks propagate preferentially along grain boundaries, but what happens exactly at the atomic scale remains unclear. Here, the authors combine atomic-scale observations and first-principles calculations to show local coordination chemistry at a dopant-segregated alumina grain boundary core dictates the atomic bond-breaking path.

## Introduction

Fracture in ceramics always limits the materials’ capability for practical applications. In many ceramics, grain boundaries behave as preferential paths for crack propagation, and thus grain boundary fracture (intergranular fracture) is a critical problem on the mechanical reliability of materials. Now, the elastic–brittle Griffith’s theory^1^ has become a fundamental theory to describe brittle fracture properties. In this theory, the external work necessary to cleave an interface is equal to the energy difference between the interface and the resultant two fracture surfaces. Although Griffith’s theory regards the initial (unfractured grain boundary) and final (two fracture surfaces) structures as two independent states, the initial and final structures are actually connected through the atomic-scale fracture process; the final fracture surfaces vary depending on the atomic fracture path inside the grain boundary core, and also the fracture path should be affected by the initial grain boundary structure^2^. However, what in the initial grain boundary determines the atomic-scale fracture path and the resultant fracture surfaces is still a matter of conjecture. In the case of fracture in ionic perfect crystals, surface charge neutrality, a surface dipole moment, the number of broken bonds, and bond valences are considered to predict the stable surfaces^3–5^. For grain boundary fractures, computational simulations such as first-principles tensile test simulations were carried out to determine the atomic fracture process so far^6–8^. However, it is unclear which fracture path is actually activated and which fracture surfaces are produced in the actual grain boundary fracture. Moreover, in real materials, foreign elements can be segregated to the grain boundary cores, as positive dopants to improve the fracture toughness or as negative impurities to worsen it^9–12^. Such complicated grain boundary core structures offer a number of possible fracture paths, and also even the chemical composition of each fracture surface depends on the path.

A robust way to determine a grain boundary fracture path will be to directly observe atomic structures of the initial grain boundary and fracture surfaces, and compare them each other. Now, (scanning) transmission electron microscopy ((S)TEM) enables to directly observe grain boundary atomic structures even in the case of dopant-segregated grain boundaries^13–16^, but for fracture surfaces, the difficulty to obtain as-fractured surfaces in a thin TEM specimen has been preventing the direct characterisation of their structures. If a crack is introduced before or during TEM specimen preparation, the fracture surfaces can be severely damaged by the specimen thinning processes, such as ion milling, mechanical polishing, etc. Recently, nanoindentation experiments inside a TEM make it possible to apply microscopically controlled stress to a TEM specimen and to observe local mechanical phenomena^17–22^. Thus, using the TEM nanoindentation techniques, it should be possible to introduce a small interfacial crack into the TEM specimen in a controlled manner^23^, and directly observe the atomic structures of the as-fractured surfaces and the crack tip by STEM.

Here, we show direct observations of as-fractured surface structures along a dopant-segregated grain boundary to determine the atomic fracture path. In the present study, we used an alumina (α-Al_2_O_3_) grain boundary with the Σ13 orientation relationship as a model case (the Σ value indicates the degree of geometrical coincidence at a grain boundary^24^.). Alumina is a typical structural ceramic and exhibits grain boundary fracture^25^, and the Σ13 grain boundary was widely studied as a model grain boundary^26, 27^. Also, we selected Zr as a dopant element, which was reported to improve toughness of alumina polycrystals by the local dopant segregation along the grain boundary^12^. Using this Zr-doped Σ13 alumina grain boundary as a model for grain boundary fracture, we directly observed the atomic structures of the as-fractured surface obtained by the TEM nanoindentation techniques, and determined the atomic-scale fracture path along the grain boundary. Our observations indicate that the local coordination geometry at the grain boundary core has strong influence on the selection of the preferential atomic fracture path. The experimental ability to directly characterise the atomic-scale fracture path will shed new light on the fundamental understanding of interfacial fracture mechanisms.

## Results

### Determination of atomic-scale grain boundary fracture path

**Fig. 1:**
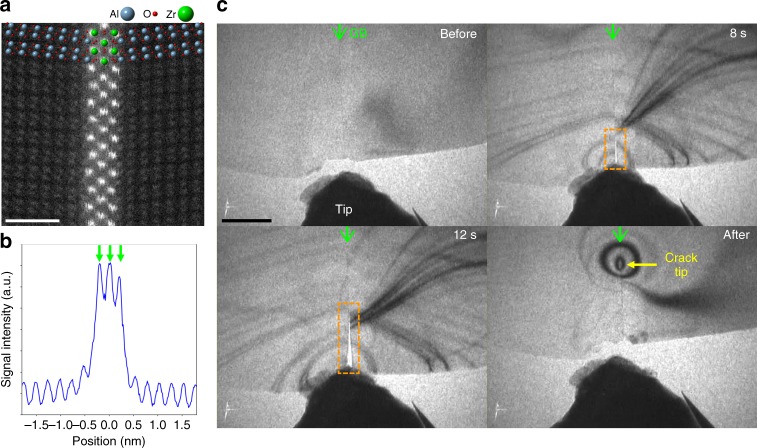
HAADF-STEM image of the Zr-doped Σ13 grain boundary and sequential TEM nanoindentation images of interfacial crack propagation. **a** HAADF-STEM image of the Zr-doped Σ13 grain boundary in alumina observed from the $\langle 2\overline {1}\overline {1}0\rangle$ directions. Zr atoms (strong intensity columns) are segregated in the triple layer at the grain boundary core. The calculated structural model is superimposed on the image, where Al, O, and Zr atoms represent as blue, red, and green colour, respectively. **b** Intensity profile of the HAADF-STEM image across the grain boundary. The signal is averaged along the vertical direction to reduce the scanning noise. Three large peaks indicated by the green arrows correspond to the triple Zr layer positions. **c** Sequential TEM images during the TEM nanoindentation experiment. The TEM movie during the nanoindentation is available in Supplementary Movie 1. The indicated times in each image represent the elapsed time after contacting the indenter tip with the specimen edge. The green arrows in each image indicate the position of grain boundary (GB), and the dashed rectangles indicate the grain boundary crack. The yellow arrow in the bottom-right image indicates the crack tip position after nanoindentation. The scale bars in **a** and **c** are 1 and 200 nm, respectively

In order to obtain the well-defined specimen, we first fabricated the bicrystals including the Zr-doped Σ13 grain boundary by thermal diffusion bonding^14–16, 27^. The bicrystallographic orientation relationship is summarised as follows: (0$$\overline {1}$$14) || (0$$\overline {1}$$14), [2$$\overline {1}$$$$\overline {1}$$0] || [$$\overline {2}$$110], and [02$$\overline {2}$$1] || [0$$\overline {2}$$21]. Figure 1a shows a high-angle annular dark-field (HAADF) STEM image of the unfractured Zr-doped grain boundary. It was found that the dopant Zr atoms are locally segregated in the triple layer at the grain boundary core. In order to determine the grain boundary structure, we constructed the structural model based on the observations, and carried out structural optimisation by the first-principles density-functional calculations (see Supplementary Note 1). The calculated structural model is superimposed on the HAADF-STEM image. The calculated grain boundary has mirror symmetry with respect to the centre Zr layer, and it agrees well with the experimentally observed structure. In the calculated structure, each Zr layer has the same Zr density, which is also consistent with the experimental image where each Zr layer has similar image intensity as shown in the intensity profile across the grain boundary (Fig. 1b). Using this grain boundary specimen, we performed nanoindentation experiments inside a TEM to introduce a crack along the segregated grain boundary. In the TEM nanoindentation experiments, we inserted the indenter tip to the grain boundary at the specimen edge, and applied local stress just on the grain boundary. Figure 1c shows the sequential TEM images captured from a movie during the nanoindentation experiments. (The movie is available in Supplementary Movie 1.) With the insertion of the indenter tip, a crack was introduced from the specimen edge, and propagated along the grain boundary plane indicated by the rectangles. When the crack reached a length of ~300 nm, we extracted the indenter tip. In the nanoindentation experiments, we successfully introduced the grain boundary crack in a controlled manner, and obtained both as-fractured surfaces and crack tip in the TEM specimen. We next observed the atomic structure of the fracture surface and the crack tip by STEM.

**Fig. 2:**
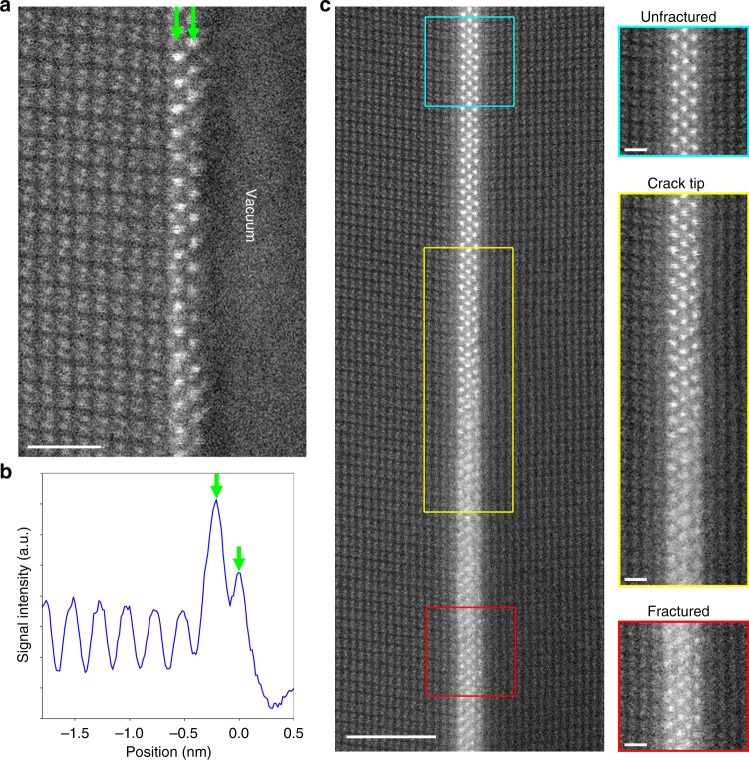
HAADF-STEM images of the fracture surface and the crack tip. **a** HAADF-STEM image of the fracture surface. The two Zr layers indicated by the green arrows remain on the fracture surface. The scale bar is 1 nm. **b** Intensity profile of the HAADF-STEM image along the horizontal direction. The signal is averaged along the vertical direction. The green arrows indicate the position of the outermost layer and the inner layer. The intensity of the outermost Zr layer becomes lower than the inner layer. **c** HAADF-STEM image of the introduced crack tip. The scale bar is 3 nm. Enlarged views of the upper (blue), middle (yellow), and lower (red) parts are also shown in the right side. The scale bars in the enlarged images are 0.5 nm

Figure 2a shows a HAADF-STEM image of the fracture surface of the left crystal. The two Zr layers were observed along the fracture surfaces. Compared with the initial triple-layered grain boundary structure, the Zr atoms in the left layer and the centre layer is considered to remain on the fracture surface of the left crystal. However, the intensity of the outermost layer of the fracture surface is significantly lower relative to the inner layer (Fig. 2b). Since the image intensity has basically positive correlation with the atomic number and the column density in a HAADF-STEM image^28^, the intensity decrease indicates that the Zr column densities in the outermost layer should be lower than that in the inner layer. Considering that each Zr layer has similar intensity in the initial structure (Fig. 1b), some Zr atoms at the each column in the initial centre layer may be also attached to the fracture surface of the right crystal in addition to the initial right Zr layer. In this experiment, we could not observe the detailed fracture surface structure of the right crystal due to the large specimen bending. Instead, we observed the crack tip to confirm the fracture path. Figure 2c shows a HAADF-STEM image of the crack tip area. In the upper part of the image (blue rectangle), the Zr atoms form the triple layer structure similar to the initial (unfractured) grain boundary structure. Going down to the middle of the image (yellow rectangle), the contrasts of the Zr columns gradually become ambiguous, and the grain boundary starts to be cleaved into the two surfaces. In the lower part (red rectangle), the Zr columns seem to form a four-layered structure. This indicates that the triple layer structure was cleaved into two layers on each fracture surface. Thus, the fracture path proceeded within the centre layer of the triple Zr layer of the initial grain boundary core, and the Zr atoms at the centre layer column are separated and attached on both fracture surfaces of the two crystals.

**Fig. 3:**
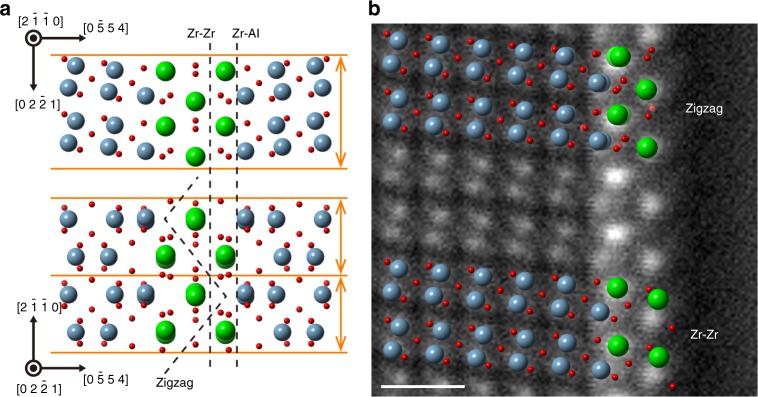
Fracture path models for the cleavage energy calculations and averaged HAADF-STEM image of fracture surface with surface structural models after relaxation. **a** The grain boundary structures viewed from two orthogonal directions. The three fracture paths (dashed lines) were selected for the calculations. The orange horizontal lines represent the structural period of the initial grain boundary. **b** Averaged HAADF-STEM image from the Fig. 2a. The two relaxed surface structural models obtained by the calculation are superimposed on the image. The upper model is for the zigzag fracture model, and the lower is for the Zr–Zr straight fracture model. The zigzag fracture model agrees with the image better than the Zr–Zr straight fracture model. The scale bar is 0.5 nm

From the comparison of these experimental results with the initial grain boundary structure, the detailed atomic-scale fracture path can be suggested. Figure 3a shows the structural model of the Zr-segregated grain boundary projected to two orthogonal directions. For ionic crystal, a stable surface should have a charge neutral surface layer or no dipole moment perpendicular to a surface^3^. If the grain boundary is cleaved along a straight path at one interatomic plane within the grain boundary core, the two fracture paths, termed the Zr–Al straight path and Zr–Zr straight path, are possible to make nonpolar surfaces. These two models, however, cannot account for the experimental results, which suggest that the fracture proceeded within the centre Zr layer and the Zr atoms at each column in the initial centre layer were attached to the two fracture surfaces. In order to make the atomic fracture path consistent with the experimental results, we have to consider the fracture path in at least twice as large structural period of the initial structure along the $\langle2\overline{1}\overline{1}0\rangle$ direction. Among the atomic fracture paths in the twice larger structural period, the zigzag fracture path as illustrated in Fig. 3a is able to divide the Zr atoms at each column in the initial centre layer into both fracture surfaces and keep the number of breaking bonds to the minimum necessary. Thus, the zigzag fracture path would be activated in the fracture of this grain boundary.

### Energetic and structural comparison with calculations

In order to verify the zigzag fracture path from the energetic aspects, we evaluated the cleavage energy of this grain boundary by first-principles density functional calculations. In this study, we used the ideal work of separation as the cleavage energy. The ideal work of separation represents the energy difference between a grain boundary and two fracture surfaces. This corresponds to the energy to cleave the grain boundary in a reversible process, and a lower bound of the external work. In practice, another irreversible work due to the discrete rupture of atomic bonds at the crack tip will be included in addition to the ideal work of separation, which is called as the lattice-trapping (or bond-trapping) effect^29–31^. In order to directly calculate the actual work of the fracture, it is necessary to perform the first-principles molecular dynamics simulations of the crack tip propagation along the grain boundary using a quite large calculation cell. Here, we used the ideal work of separation here as a first approximation to compare the energy cleavage^32, 33^. The fracture models of the Zr–Al straight path and Zr–Zr straight path, as well as the zigzag fracture path model were also evaluated for comparison. The calculated supercells are described in Supplementary Fig. 2. The calculations show that the zigzag fracture produces the minimum energy of 2.66 J m^−2^, while the Zr–Al straight fracture model and the Zr–Zr straight fracture model have the energies of 4.66 and 3.61 J m^−2^, respectively. Thus, the zigzag fracture path is the most stable path in the three models from the present calculations. Moreover, the observed fracture surface structure is consistent with the calculated surface structure for the zigzag model. Figure 3b shows the averaged STEM image of the fracture surface with the surface structural models after the structural relaxation of the surface atoms. The relaxed surface structure of the zigzag fracture model agrees well with the image, while the relaxed surface structure of the Zr–Zr straight fracture model differs from the image at the Zr columns in the outermost layer. Thus, the theoretical calculations also support the zigzag fracture path in both energetic and structural aspects.

## Discussion

**Fig. 4:**
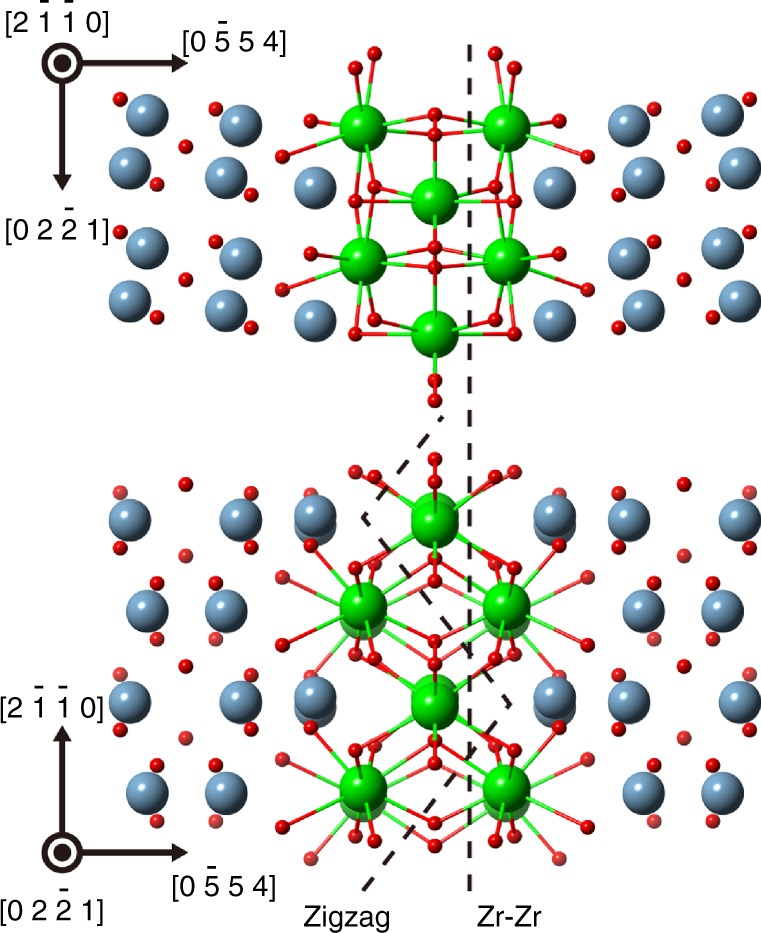
Zr–O bonds cut along the zigzag fracture path and the Zr–Zr straight path. The structural model of the grain boundary viewed from two orthogonal directions with Zr–O bonds. In the initial structure, Zr atoms take oxygen atoms as eight-fold coordination. In both zigzag and Zr–Zr straight paths, Zr–O bonds broken in the fracture are equivalent and the same numbers, but the oxygen coordination numbers of Zr on fracture surfaces are different in both paths

Structurally, in both the zigzag fracture path and the Zr–Zr straight fracture path, the same number of Zr–O bonds should be cut during the fracture processes. Moreover, considering the structural mirror symmetry with respect to the centre Zr layer, the Zr–O bonds broken in the two models are equivalent as shown in Fig. 4. Thus, the broken bond characters such as bond length or bond valence should be the same for both paths. However, the zigzag path is experimentally observed and also theoretically favourable fracture path. From the viewpoint of the local oxygen coordination numbers, all Zr atoms at the initial grain boundary take eight-fold coordination. In the Zr–Zr straight fracture shown in Fig. 4, 8 six-fold coordinated Zr are produced on the two fracture surfaces. On the other hand, the zigzag fracture produces 4 six-fold coordinated Zr and 8 seven-fold coordinated Zr. In zirconium oxide, Zr atoms takes oxygen atoms as seven-fold or eight-fold coordination, and it will need relatively higher energy to loss oxygen coordination into the six-fold coordination^34^. Thus, the zigzag fracture which produces much less coordination-deficient oxygen polyhedra than the straight case may be therefore selected as the preferable fracture path of the grain boundary. These results indicate that the local atomic coordination chemistry of the segregated cation in grain boundary core will affect the preferable fracture path selection and the resultant fracture surface structures.

The present study demonstrates the experimental determination of atomic-scale fracture path within dopant-segregated grain boundary by using the TEM nanoindentation techniques combined with the atomic-scale STEM imaging techniques. The present findings indicate that the local geometry of coordination polyhedra at the dopant-segregated grain boundary core should determine the atomic fracture path and fracture surfaces, adding to a surface dipole moment and the number of breaking bonds. The ability to directly characterise the atomic-scale fracture path brings a crucial step towards the fundamental and quantitative understanding of interfacial fracture mechanisms in materials.

## Methods

### Specimen preparation

The Al_2_O_3_ bicrystal was fabricated by the thermal diffusion bonding of two high-purity single crystals (>99.99%, Shinkosha Co. Ltd.) at 1500 °C for 30 h in air. For doping Zr, we spattered Zr on the single crystal surface before bonding using an etch-coating system (Model 682, Gatan Inc.). The specimens for TEM observations were prepared as follows. We cut the fabricated bicrystals into small pieces and polished mechanically to a thickness of about 20 μm. They were further thinned to electron transparency by Ar^+^ ion milling using an ion polishing system (Model 691, Gatan Inc.).

### TEM nanoindentation and STEM observation

The TEM nanoindentation experiments were performed inside a JEM-2010 microscope (JEOL Ltd.) operated at 200 kV using a double-tilt TEM-nanoindenter holder (Nanofactory Instruments AB), and the STEM observations were carried out using a JEM-ARM200 microscope (JEOL Ltd.) operated at 200 kV equipped with an aberration corrector and a cold field emission gun. The objective lens of the JEM-2010 microscope has wide polepiece gap (high-contrast type polepiece), and the nanoindentaer holder with large physical size can be compatible with the JEM-2010. The detailed experimental procedures for the TEM nanoindentation experiments are described elsewhere^21^. In the nanoindentation experiments, we manually inserted a wedge-shaped indenter tip made of diamond. The indentation movies were recorded with a frame rate of 30 fps using a video camera for TEM and a video recorder (SR-DVM700, Victor Ltd.).

### First-principles calculations

The first-principles calculations were performed using the VASP code. The calculation conditions and the calculated supercell to obtain the initial grain boundary structure are described in Supplementary Note 1 and Supplementary Fig. 1. For the cleavage energy calculations, the conditions and the supercells are shown in Supplementary Note 2 and Supplementary Fig. 2.

## Supplementary information

Supplementary Information

Description of Additional Supplementary Files

Supplementary Movie 1

## Data Availability

The data that support the findings of this study are available from the corresponding author upon request.
