# Supplementary material for: Direct observation of atomic-scale fracture path within ceramic grain boundary core
Source: Nat Commun. 2019 May 8;10:2112. doi: 10.1038/s41467-019-10183-3 (PMC6506594; doi:10.1038/s41467-019-10183-3)
Supplement: Supplementary file 1 — Supplementary Information [file 41467_2019_10183_MOESM1_ESM.pdf]

Supplementary Information

Direct observation of atomic-scale fracture path within ceramic grain boundary core

Kondo *et al.*

Supplementary Note 1: First-principles calculations of the grain boundary structure

The structure of the Zr-doped alumina Σ13 grain boundary was calculated by the first-principles density-functional calculations. The initial structural model was constructed from the pure Σ13 grain boundary reported elsewhere[1,2] by substituting Zr atoms for Al atoms. Supplementary Figure 1a shows the atomic model of the pure Σ13 grain boundary structure. In the supercell, two equivalent grain boundaries included inside to satisfy the periodic boundary condition along the vertical direction. As shown in Supplementary Figure 1b, we substituted, for each grain boundary, the 6 Zr atoms for the Al atoms to form the triple layer structure as observed experimentally, and also introduced the 2 Al vacancies at the centre Zr layer to compensate the excessive charge as the following equation,

$$3\mathrm{Zr} \rightarrow 3\mathrm{Zr}_{\mathrm{Al}}^{'} + \mathrm{V}_{\mathrm{Al}}^{\cdots} \, . \qquad (1)$$

The total number of atoms is 196 atoms. The structural relaxation was carried out using the VASP code. The projected augmented-wave method was applied with the GGA-PBE potential. We used the *k*-points of 3×2×1 grids and the cut-off energy of 500 eV. The structural optimizations were performed for the ion positions, the supercell shape, and supercell volume, which finished with the maximum force < 0.02 eV Å^{-1}. Supplementary Figure 1c shows the structural model after relaxation, which agree with the experimental image as shown in Fig. 1a in the main text.

Supplementary Note 2: First-principles calculations of the cleavage energy

The cleavage energies were evaluated using the ideal work of separation, which is the energy difference between a bulk model and two slab models[3,4]. The bulk model includes the two equivalent grain boundaries to satisfy the periodic boundary condition of the supercell, and the two slab models were obtained by cutting the bulk model at two equivalent fracture paths for each model. To calculate the cleavage energies for three fracture models, we first prepared the bulk model (the grain boundary model) with twice larger supercell size along the $\langle 2\bar{1}\bar{1}0 \rangle$ directions (Supplementary Figure 2a). Using this model including 392 atoms in total, the energy of the bulk model (E_{bulk}) was calculated with the *k*-points of 3×2×1 grids. The other conditions are same as the above grain boundary calculation. We also prepared two slabs for each model by cutting the bulk model at equivalent fracture planes, and calculated the energies of two slabs (E_{slab1}, E_{slab2}) for the three fracture models without structural relaxations. As shown in Supplementary Figure 2b, each slab has equivalent surfaces on both sides to avoid the introduction of dipole moment to the slab. The ideal work of separation is evaluated by the following equation,

$$E_{\mathrm{cleavage}} = \frac{E_{\mathrm{slab1}} + E_{\mathrm{slab2}} - E_{\mathrm{bulk}}}{2A}, \qquad (2)$$

where A represents the area of grain boundary in the supercell, and the factor 2 accounts for two grain boundaries present within the supercell.

We also calculated the relaxed surface structures of the Zr-Zr straight fracture model and the zigzag fracture model by optimizing ion positions of the slabs. The structural models of the relaxed surfaces are superimposed on the HAADF-STEM image of the fracture surface in Fig. 3b, where the zigzag model agrees better than the Zr-Zr straight fracture model.

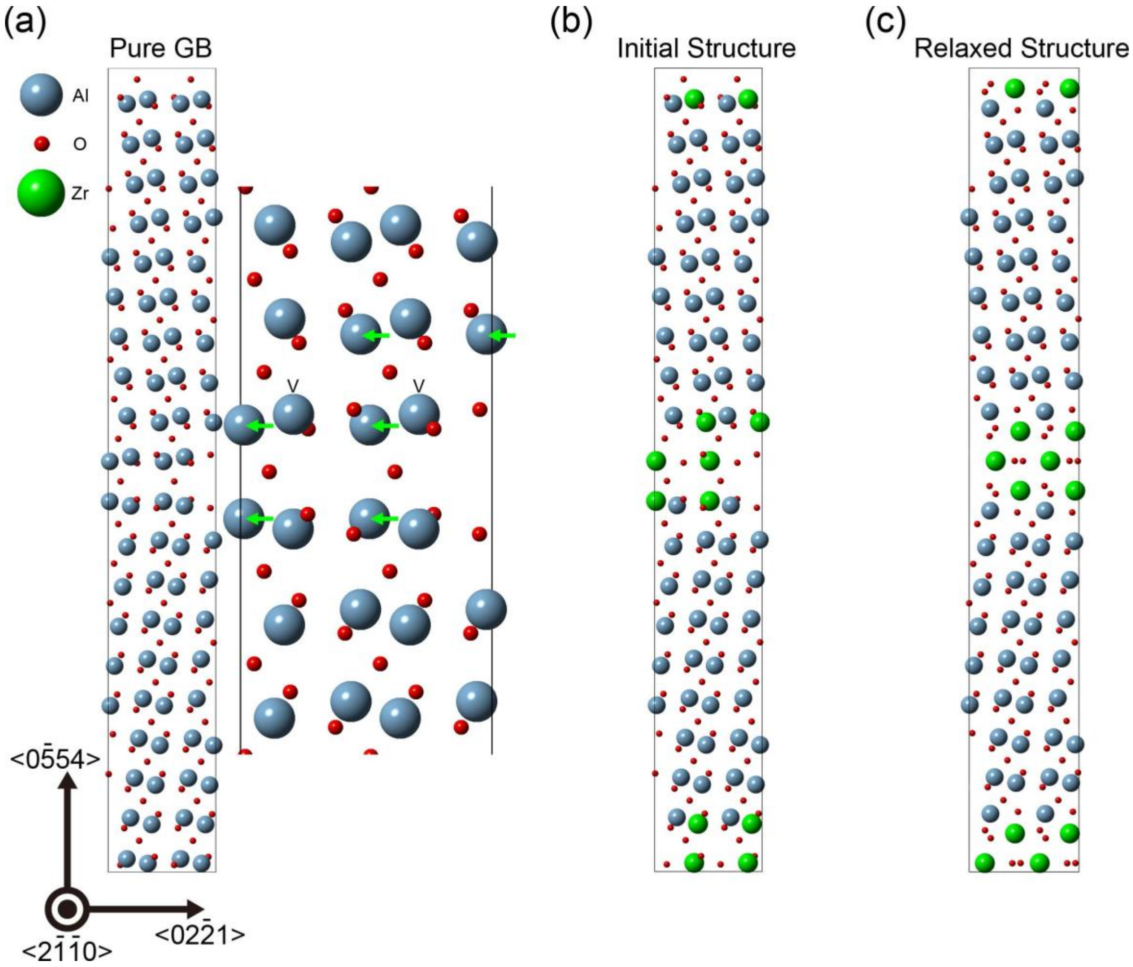

Supplementary Figure 1: First-principles calculations of the Zr-doped alumina Σ13 grain boundary. a, Structural model of the pure alumina Σ13 grain boundary. For making the Zr-doped model, the green arrows indicate substitution sites of Zr atoms while "V" denote vacancy sites. **b,** Initial model for the calculation. **c,** Structural model after relaxation.

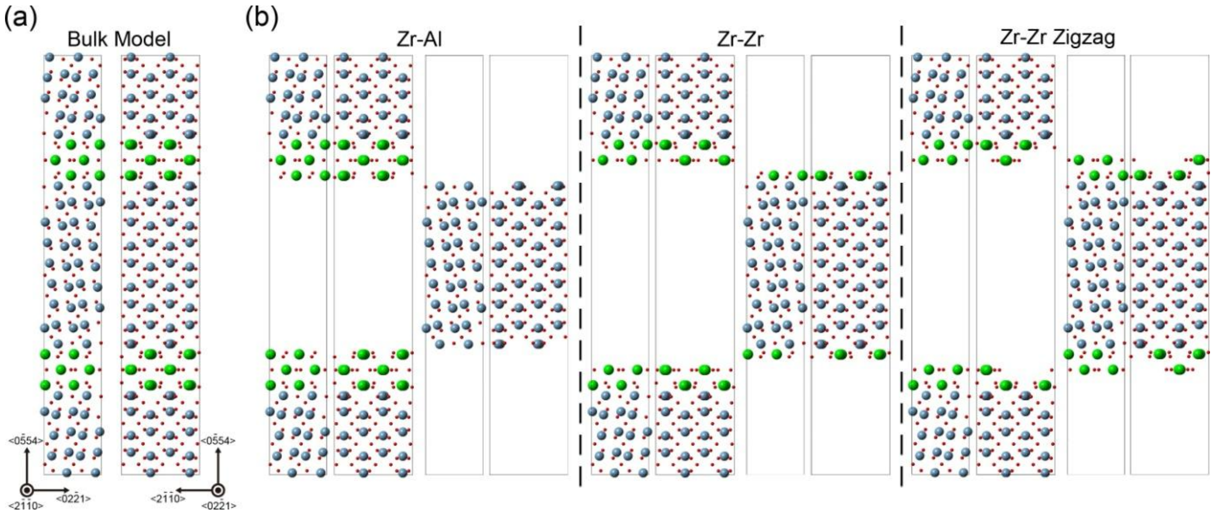

Supplementary Figure 2: Structural models for cleavage energy calculations observed from two orthogonal directions. a, Bulk model for the calculations. The supercell size to the $\langle 2\bar{1}\bar{1}0 \rangle$ directions is twice larger than the periodicity of this grain boundary. **b,** Slab models for three fracture models. The models were obtained by cutting the bulk model at two equivalent plane.

Supplementary References

1. Fabris, S. & Elsässer, C. Σ13 $(10\bar{1}4)$ twin in α-Al_2O_3: A model for a general grain boundary. *Phys. Rev. B* **64**, 245117 (2001).
2. Azuma, S. *et al.* HAADF-STEM observations of a Σ13 grain boundary in α-Al_2O_3 from two orthogonal directions. *Phil. Mag. Lett.* **90**, 539-546 (2010).
3. Batirev, I. G., Alavi, A. & Finnis, M. W. First-principles calculations of the ideal cleavage energy of bulk niobium (111) / α-alumina (0001) interfaces. *Phys. Rev. Lett.* **82**, 1510-1513 (1999).
4. Schweinfest, R., Paxton, A.T. & Finnis, M. W. Bismuth embrittlement of copper is an atomic size effect. *Nature* **432**, 1008-1011 (2004).
