# Supplementary material for: Direct observation of atomic-scale fracture path within ceramic grain boundary core
Source: Nat Commun. 2019 May 8;10:2112. doi: 10.1038/s41467-019-10183-3 (PMC6506594; doi:10.1038/s41467-019-10183-3)
Supplement: Supplementary file 2 — Description of Additional Supplementary Files [file 41467_2019_10183_MOESM2_ESM.docx]

Description of Additional Supplementary Files

**Supplementary Movie 1 | Experimental bright-field TEM movie of nanoindentation for the Σ13 grain boundary.** The movie is played with the real time speed. The captured images from this movie are presented in Fig. 1c in the main text.
